# Society for the Prevention and Relief of Cancer

**Published:** 1919-11-08

**Authors:** Douglas Macmillan

**Affiliations:** Honorary Secretary.


*To the Editor of* THE HOSPITAL.

### Society for the Prevention and Relief of Cancer.

*To the Editor of* THE HOSPITAL.

SIR,—My attention having been directed to your issue dated the 1st inst., I may perhaps be permitted to thank you for publishing a notice of a pamphlet ("Cancer Research and Vivisection") recently published by this Society, although I can hardly compliment you upon the tone of it. It would almost appear that, instead of attempting to give a fair idea of the contents and spirit of the book—the main contention of which he admits to be true—your reviewer jumps at the opportunity of attacking a philanthropic Society of which he appears to know nothing, but of which he could easily have obtained the fullest information. The fact that an effort of this kind is controlled principally by laymen is surely not sufficient offence for it to be condemned—by a responsible journal—to the lay public, who, after all, are the principal supporters of all such movements, and, incidentally, the principal sufferers from an unconquered scourge.

I enclose a recent Report, with a full list of officers, of this Society, and must request you to correct your reviewer's erroneous statement that no such information is published—a statement which is quite untrue and which might be likely (I hope it is not *intended*) to damage a genuine and earnest effort for the public welfare. In its origins, objects, and management alike, my Society is open to the fullest investigation.—Yours truly,

DOUGLAS MACMILLAN,
Honorary Secretary.

[The pamphlet in question contained, as was stated in the review of which Mr. Macmillan complains, an appeal for funds, and did not disclose any information about this Society's officers beyond that mentioned. In our view, any Society issuing an appeal for funds should publish *with that appeal* sufficient information as to its aims and officers to enable the public to form some idea of how the funds will be applied. From the leaflets now sent it is possible to learn that one of the Vice-Presidents of Mr. Macmillan's Society is Miss Lind-af-Hageby, and that another is a Vice-President of the British Anti-Vivisection Society. It is noticeable also that Mr. Macmillan quite omits any disclaimer of hostility to vivisection. From this the conclusion suggested in THE HOSPITAL as possible—that this Society is anti-Vivisectionist in aim and tone—appears more than ever justified. As regards the control of this Society by laymen, there are one or two observations that seem to be pertinent. A society which aims at the "Prevention and Relief of Cancer" without the co-operation of a single medical man of recognised eminence in his profession can scarcely expect to be taken seriously. The problem which cancer presents is so serious and so difficult that only the best scientific brains of the country should be encouraged to tackle it. Any scheme which neglects this principle is merely wasting labour and money. It is true that the S.P.R.C. can hardly be accused of wasting much money, inasmuch as its total income for 1918 is given by Mr. Macmillan as £48, and the total expenditure as £21. But the Society professes to have as one of its "foremost objects" the provision of hospital accommodation for the treatment of cancer, and to establish "not one, but several cancer hospitals in various parts of the country"; and it appeals to the wealthy and philanthropic for donations and legacies. With the fuller information now available, by Mr. Macmillan's courtesy, we can only reiterate with much more conviction and energy than before that the S.P.R.C. cannot be recommended to public support.—ED. THE HOSPITAL.]

